# Metabolic control analysis of L-tryptophan producing *Escherichia coli* applying targeted perturbation with shikimate

**DOI:** 10.1007/s00449-021-02630-7

**Published:** 2021-09-14

**Authors:** Kristin Schoppel, Natalia Trachtmann, Fabian Mittermeier, Georg A. Sprenger, Dirk Weuster-Botz

**Affiliations:** 1 Institute of Biochemical Engineering, Technical University of Munich, Boltzmannstraße 15, 85748 Garching, Germany; 2 Institute of Microbiology, University of Stuttgart, Allmandring 31, 70569 Stuttgart, Germany

**Keywords:** Metabolic control analysis, L-tryptophan, *Escherichia coli*, Thermodynamics-based flux analysis, Shikimate

## Abstract

L-tryptophan production from glycerol with *Escherichia coli* was analysed by perturbation studies and metabolic control analysis. The insertion of a non-natural shikimate transporter into the genome of an *Escherichia coli* L-tryptophan production strain enabled targeted perturbation within the product pathway with shikimate during parallelised short-term perturbation experiments with cells withdrawn from a 15 L fed-batch production process. Expression of the shikimate/H^+^-symporter gene (*shiA*) from *Corynebacterium glutamicum* did not alter process performance within the estimation error. Metabolic analyses and subsequent extensive data evaluation were performed based on the data of the parallel analysis reactors and the production process. Extracellular rates and intracellular metabolite concentrations displayed evident deflections in cell metabolism and particularly in chorismate biosynthesis due to the perturbations with shikimate. Intracellular flux distributions were estimated using a thermodynamics-based flux analysis method, which integrates thermodynamic constraints and intracellular metabolite concentrations to restrain the solution space. Feasible flux distributions, Gibbs reaction energies and concentration ranges were computed simultaneously for the genome-wide metabolic model, with minimum bias in relation to the direction of metabolic reactions. Metabolic control analysis was applied to estimate elasticities and flux control coefficients, predicting controlling sites for L-tryptophan biosynthesis. The addition of shikimate led to enhanced deviations in chorismate biosynthesis, revealing a so far not observed control of 3-dehydroquinate synthase on L-tryptophan formation. The relative expression of the identified target genes was analysed with RT-qPCR. Transcriptome analysis revealed disparities in gene expression and the localisation of target genes to further improve the microbial L-tryptophan producer by metabolic engineering.

**Supplementary Information:**

The online version contains supplementary material available at 10.1007/s00449-021-02630-7.

## Introduction

Metabolic engineering has been widely used to reroute fluxes towards the desired products to overcome production limitations of biotechnological processes due to constraints and regulations in cell metabolism. However, rational design is impeded by the complexity of biosynthesis pathways, and in vitro analyses of single enzymes cannot display processes in vivo realistically. Perturbation experiments have opened up the possibility of characterising metabolic pathways in vivo during microbial production processes [1]. Termed ‘metabolic analyses’, these experiments allow detailed insights into the cells’ metabolism and generate kinetic information on the enzymatic network.

Stationary metabolic analyses are one kind of metabolic analyses and they are based on the deflection of a reference state by either change in substrate or substrate availability [2–4]. As a reaction to the disturbance, the metabolism will be shifted, and a new metabolic steady state can be established within minutes inside the cells [1, 5]. Rapid Media Transition is one option for conducting metabolic analyses [4]. Interesting time points in continuous or semicontinuous fed-batch processes serve as reference states from which cell broth is sampled, centrifuged and rapidly transferred with fresh media into smaller stirred-tank bioreactors used for short-term perturbation studies. Disturbance of the reference state can be avoided by separation from the process reactor [6]. The parallelisation of stirred-tank bioreactors for metabolic analyses increases the volume of data generated. Perturbation of cell metabolism is achieved by supplying different carbon sources [4] or by varying the substrate supply rates [2]. Dynamic changes in intra- and extracellular metabolite concentrations are captured by rapid sampling [5] during the cell deflection.

To enable targeted genetic modifications of production strains, metabolic control analysis (MCA) is applied. The mathematical method allows the identification of rate-limiting steps within the biosynthesis pathway [7]. Prerequisite for MCA is a complete metabolome dataset and a thermodynamic analysis for the considered metabolic network, to derive information on displacement from reaction equilibrium of contributing reactions in the model. Due to technical difficulties during the experimental determination of intracellular metabolite concentrations, where artefacts occur, or analytical platforms have limitations [8, 9], the acquisition of metabolome data can be incomplete. To deduce the missing information and calculate thermodynamic quantities, ‘network-embedded thermodynamic analysis’ (NET-analysis) methods were developed. These methods apply the second law of thermodynamics, the metabolites’ Gibbs energies of formation, reaction directionalities and stoichiometries to connect metabolite concentrations to their metabolic networks [8]. The outputs are feasible ranges for Gibbs energies of reactions within the chosen model and missing metabolite concentration ranges [8].

Furthermore, the implementation of MCA requires information on intracellular reaction rates. Fluxome data, derived from perturbation experiments, is used to compute intracellular flux distributions via constraint-based approaches like Flux Balance Analysis or Flux Variability Analysis [10]. Methods like ‘thermodynamics-based flux analysis’ (TFA) [11–13] enable the restriction of constraint-based flux analysis of genome-scale models with thermodynamics, avoiding flux distributions that contradict cell physiology and thermodynamics of metabolic processes [11, 12, 14].

Compared to NET-analysis, TFA is beneficial since the flux solution space is not biased concerning reaction directionalities [13]. A Python and MATLAB implemented version of TFA combines thermodynamic calculations and flux analyses in one mixed-integer linear programming problem in consideration of intracellular metabolomic data [14].

Aromatic amino acids are an example of a group of biochemicals where production capacities and economic efficiency by fermentation remain low, whereas market demand steadily increases [15, 16]. Hence, the method of parallelised metabolic analysis with rapid media transition was successfully applied to L-phenylalanine production with *E. coli* from glycerol [2]. As L-tryptophan production, in particular, still lags behind market demand [15, 16], the method has been transferred so that L-tryptophan production from glycerol can be investigated in detail [17].

For humans and livestock, L-tryptophan is an essential amino acid and they are dependent on consuming it with nutrition [18]. Thus, one major field of application is the supplementation of food and animal feed [15]. L-tryptophan is also applied for pharmaceutical purposes as a precursor metabolite for several compounds in human and animal metabolism. For instance, it acts as an immediate precursor of serotonin and can therefore be used as a mild antidepressant and insomnia medication [15]. The most relevant producer strains for L-tryptophan are *Escherichia coli* (*E. coli)* and *Corynebacterium glutamicum*. However, biosynthetic L-tryptophan production is energetically expensive and to prevent cells from wasting energy, it is controlled by strict regulation on multiple levels, including feedback inhibition, attenuation and repression [18–22]. To optimise producer strains it is aimed to circumvent these regulatory mechanisms, and a multitude of possible targets can be developed for rational design. Possible modifications to improve L-tryptophan producer strains have been previously summarised by [18, 20, 22–26]. High L-tryptophan concentrations of 48.68 g L^−1^ with glucose as carbon source were reported with rationally engineered *E. coli* strains [23], and increased titres of 52.57 g L^−1^ were reached in combination with adapted pH and dissolved oxygen control strategies in the fed-batch process [27]. Aside from high product concentrations, the economic efficiency of production processes is also dependent on substrate conversion rates and the current market values of the carbon sources used. In terms of sustainability and recycling, glycerol is an alternative resource to sugars such as glucose. This is because a considerable amount of crude glycerol has become available as a waste stream through the accelerated development of biodiesel production over the last decades [28]. The resulting excess of glycerol has caused a sharp decrease in prices, and the conversion of the alternative carbon source into valuable products has become economically attractive [28–30]. There are some distinct advantages regarding sustainability, as glycerol production does not compete with food industry [29, 31]. For microbial usage, glycerol enables potentially increased product yields, due to glycerol’s higher degree of reduction compared to sugar’s [29, 30, 32].

In *E. coli* cells, glycerol is taken up either via passive diffusion or through protein-assisted facilitated diffusion [33, 34]. This circumvents the competitive metabolization of phosphoenolpyruvate (PEP) between phosphotransferase system (PTS) and reactions in the biosynthesis pathway of aromatic amino acids, as occurs when glucose is the carbon source [35]. Despite several theoretic advantages of glycerol usage, only a few production processes for making aromatic compounds exist, and product yields remain low compared to processes based on glucose [36–38]. An L-tryptophan production process with glycerol was recently established, leading to 12.5 g L^−1^ L-tryptophan [37]. L-tryptophan concentrations of 14.3 g L^−1^ were obtained by further strain optimisation [17]. Detailed investigation of the cell metabolism during L-tryptophan production by parallelised metabolic analysis with subsequent MCA indicated that limitations are also located in the aromatic amino acid pathway and particularly in L-tryptophan biosynthesis pathway [17]. Indol-glycerolphosphate synthase and tryptophan synthase have been identified as limiting enzymatic steps within L-tryptophan biosynthesis [17].

The accuracy of these results is strongly dependent upon the degree of deflection, which is induced by the applied perturbation substrates. The variety of substrates for metabolic analysis is limited to a narrow selection of carbon sources, which can be taken up naturally by the cells under examination. Most perturbation experiments are performed with glucose; other standard perturbation substrates are glycerol, acetate, succinate, pyruvate or ethanol [1, 2, 4, 39–46]. Even though perturbation with these substrates leads to deflections in fluxes and intracellular metabolite concentrations within the aromatic amino acid pathway [2, 17], the more downstream a pathway intermediate is located, the less its concentration is affected by changes in substrate availability [47]. An enhancement of deviations within the product pathway could improve the predictive significance of MCA results for the pathways considered. To select suitable perturbation substrates, the proximity of the entry point to the considered product pathway is of importance, favourable is a natural uptake system for the desired substrate and commercial availability is determinant for the applicability of potential substrates in metabolic analyses. In previous studies, a transporter strain has been successfully applied to enable target-specific perturbation at specific points in the metabolism with PEP as a non-natural substrate and as effector, without influencing the production process, to evade substrate limitations [35].

**Fig. 1:**
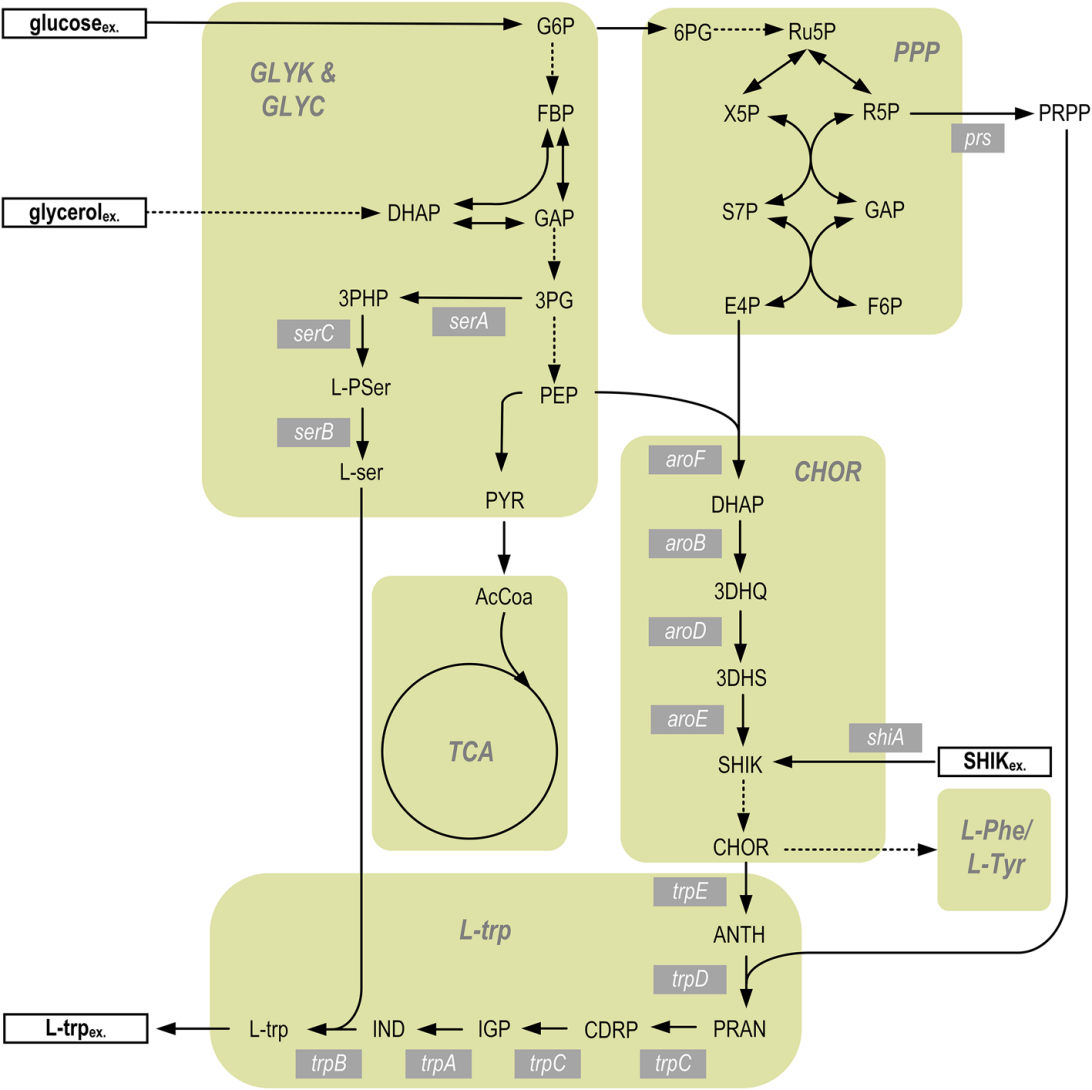
Schematic representation of central carbon metabolism and L-tryptophan biosynthesis pathway with the marked point of entry for shikimic acid. Depicted are the metabolites glucose-6-phosphate (G6P), fructose 1,6-bisphosphate (FBP), glyceraldehyde 3-phosphate (GAP), 3-phospho-D-glycerate (3PG), phosphoenolpyruvate (PEP), pyruvate (PYR), 3-phosphohydroxypyruvate (3PHP), O-phospho-L-serine (L-PSer) and L-serine (L-ser) from glycolysis and glycerol metabolism (*GLYC & GLYK*), 6-phosphoglucono-1,5-lactone (6PG), ribulose 5-phosphate (Ru5P), xylulose 5-phosphate (X5P), ribose 5-phosphate (R5P), sedoheptulose 7-phosphate (S7P), erythrose 4-phosphate (E4P), fructose 6-phosphate (F6P) and 5-phospho-alpha-D-ribose 1-diphosphate (PRPP) from pentose-phosphate-pathway (*PPP*), Acetyl-Coa (AcCoA) from citric acid cycle (*TCA*), chorismate (CHOR), 3-dehydroquinate (3DHQ), 3-dehydroshikimate (3DHS), shikimate (SHIK) and chorismate (CHOR) from chorismate biosynthesis pathway (*CHOR*) as well as anthranilate (ANTH), N-(5-phospho-D-ribosyl)anthranilate (PRAN), 1-(2-Carboxyphenylamino)-1-deoxy-D-ribulose 5-phosphate (CDRP), 3-Indolyl-glycerol 3-phosphate (IGP), indole (IND) and L-tryptophan (L-trp) from L-tryptophan biosynthesis pathway (*L-trp*). Relevant genes are denoted in a white font with grey background

Shikimic acid is an intermediate metabolite in the common aromatic amino acid pathway. Due to its central position in chorismate biosynthesis and proximity to the L-tryptophan biosynthesis pathway, it is a promising substrate for perturbation during the metabolic analysis of L-tryptophan production. Its position in *E. coli* metabolism is depicted in Fig. 1. Although wild-type *E. coli* carries a *shiA* gene, encoding a shikimate/ H^+^-symporter, it shows relatively poor ability to importshikimic acid [48]. *ShiA* gene from *E. coli* has been characterised and cloned by Whipp [49], and introducing *shiA* on a multicopy plasmid, enabled the growth of *E. coli* with shikimate. Kubota [50] first identified a *shiA* gene in the Gram-positive *C. glutamimcum* used in this study to express a shikimate transporter to a sufficiently high extent so that an enhanced deflection of fluxes and metabolome within the pathway of aromatic amino acids during parallelised metabolic analysis can be achieved. MCA is performed to reveal the control inside the cells, during L-tryptophan production. As new approach, the recombinant shikimate transporter strain was engineered, which enabled the usage of shikimic acid as additional perturbation substrate during metabolic analysis, to reach a highly targeted perturbation within the pathway of aromatic amino acid biosynthesis. To improve the validity of flux analyses and MCA, we combined the extensive dataset derived from metabolic analysis with a recently published thermodynamics-based flux analysis by Salvy [14].

## Material and methods

### Strains, plasmids and DNA primers

All plasmids, strains and primers used in this paper are listed in Table 1. The NT1259*shiA*_Cg_ strain was constructed using the CRISPR-Cas-mediated method as described earlier by Jiang et al.[51]. The plasmid pTarget-_sgMal_ was developed by inverse PCR with the “sg-RNA -mal” and “sg-reverse” primers (see Table 1). The fragment containing P_tac_-*shiA*_*Cg*_ (*shiA*_Cg_ encodes the shikimate transporter from *Corynebacterium glutamicum* R strain [50]) flanked with homology regions (about 40 nt) was amplified from pEKEx-P_tac_-*shiA*_cg_ plasmid with “malE-int” and “malG-int” primers. The PCR-fragment was introduced directly into the chromosome of the NT1259 strain by homology recombineering using the λ-Red system, cloned on the pCas plasmid [51].

**Table 1:** Strains and plasmids used in this study

Strain		Genotype	Source or reference
NT1259		W3110 Δ*trp*L *trp*E ^FBR^ (M293) N168D A478T C237R Δ*trp*R:FRT Δ*tna*A::FRT Δ*sda*B::FRT Δ*lac*:: P_tac_-*aro*F-B-L Δ*fuc*::P_tac_-*tkt*A Δ*xyl*::P_tac_-*ser*A^FBR^ (T372N) Δ*rib*::P_tac_-*trp*B-*trp*A	[37]
NT1259 shiA_Cg_		NT 1259 as above ∆*mal*::*shiA*Cg	This work
*Plasmid*			
pCas	*RepA101*(Ts) *kan P*_*cas*_*-Cas9 P*_*araB*_*-Red lacI*^*q*^* P*_*trc*_*-*sgRNA*-pMB1*		[51]
pTarget-cat	*pMB1 cat*		[37]
pTarget_sgMal_	*pMB1 cat* sgRNA*-Mal*		This work
pEKEx-P_tac_-shiACg_*cg*_	*kan*^*R*^*; lacI*^*q*^* pBL1 oriV*_*Cg*_* pUC18 oriV*_*Ec*_*;P*_*tac*_*-shiA,*		[50] (J-W Youn, pers. commun)
pF*aro*FBL_Kan_	*kan*^*R*^* colE lacI*^*q*^* P*_*tac*_*-aroFBL*		[37]
*Primers*			
malE-int	AAGGTAAACTGGTAATCTGGATTAACGGCGATAAAGGCTATAACGGTCTCGCTGTCAAGGCGCACTCCCGTTCTGG		
malG-int	GATCGGTAATGCAGACATCACGGCAGCGGCGGCAAAGTCACCCCACAGGTAGTTTTCAGGGTTATTGTCTCATGAGCG		
sgRNAmal	GTTGCCATCCGGCAGAATGGGTTTTAGAGCTAGAAATAGCAAGTTAAAATAAGGCTAG		
sg-reverse	ACTAGTATTATACCTAGGACTGAGCTAGC		
RT-serB-5'	AAGTAACCGAACGGGCGATGCG		
RT-serB-3'	CCGGAGGCAATCGCCACTTTC		
RT-serA-5'	CAAGATTAAGTTTCTGCTGGTAGAAGGCG		
RT-serA-3’	TAGCGACCAGTTTTTCTGCGGCGTTG		
RT-prs-5’	ACAACGCCGCCGATGTCCGGAG		
RT-prs-3'	ACTGCGAAAGTGGTTGCAGACTTCC		
RT-aroB-5’	GCATGGTCGCGATACTACGCTGG		
RT-aroB-3’	CGCGCCAATCATGTTTTTACCGAGG		
RT-aroF-5’	GCTGCAGATCGCGCGTAAATTGCTG		
RT-aroF-3’	CCGGAGGCCATTTCACGGTGAG		
RT-trpC-5’	CTTATGACGCGGGCGCGATTTACGG		
RT-trpC-3’	CCAGCGATAACACCTTAGCTTTGTCC		
RT-trpB-5’	GTTTTACCCATCCGCTTCGCCAGCAAC		
RT-trpB-3’	AATTTCAGGCTCAGTTCAACGACCTGCTG		
RT-ftsZ-5’	TGCATTTGCTTCCGACAACG		
RT-ftsZ-5’	ACGTTTGTCCATGCCGATAC		

### Growth medium and inoculum preparation

A defined minimal medium, adapted from Albermann [52], was used to cultivate *E. coli* NT1259 *shiA* pF*aroFBL*_Kan_ in preculture, the production process, and metabolic analysis. Heat-resistant components (g L^−1^): 3.00 KH_2_PO_4_, 12.00 K_2_HPO_4_, 5.00 (NH_4_)_2_SO_4_, 0.10 NaCl, were dissolved in water and autoclaved, heat-labile ingredients (g L^−1^): 0.30 MgSO_4_·H_2_O, 0.015 CaCl_2_·2 H_2_O, 0.1125 FeSO_4_·7 H_2_O, 1.50 sodium citrate, 0.0075 thiamine and 0.05 kanamycin were sterile-filtrated separately and added after cooling. A stock solution of glycerol (1000 g L^−1^) was also autoclaved, and 7 g L^−1^ were added to the medium for inoculum preparation, 4 g L^−1^ to the medium for the production process. 1 mL L^−1^ of a trace-element solution [53], containing (g L^−1^) 11.2 MnSO_4_·H_2_O, 10.0 AlCl_3_·6 H_2_O, 7.3 CoCl_2_·6 H_2_O, 2.0 ZnSO_4_·7 H_2_O, 2.0 Na_2_MoO_4_·2 H_2_O, 1.0 CuCl_2_·2 H_2_O, 0.5 H_3_BO_3_, was added to the production medium.

Two individual colonies of *E. coli* NT1259 *shiA* pF*aroFBL*_Kan_ were picked from an agar plate to inoculate two 100 mL shake flasks with 20 mL minimal medium each to generate a starter culture for the fed-batch cultivation on a 15 L scale. After 40 h of incubation at 37 °C and 100 min^−1^ in an orbital shaker (Multitron incubation shaker, Infors HT, Bottmingen, Switzerland), the cell suspension was transferred into ten 500 mL shake flasks with 100 mL minimal medium each. A volume of 1.0 L of cell suspension for the inoculation of the production process was obtained after 20 h incubation at 37 °C and 250 min^−1^ orbital shaking. The emerging cell suspension was transferred into a sterile bottle and pumped aseptically into the stirred-tank reactor.

### L-tryptophan fed-batch production process on a 15 L scale

A modified fed-batch process was used to produce L-tryptophan [36, 37]. Cultivation was carried out in a 42 L stainless-steel stirred-tank bioreactor (Techfors, Infors HT, Bottmingen, Switzerland) with a maximum working volume of 30 L. Four equidistant baffles and three six-bladed Rushton impellers were used to achieve good mixing and thorough gas dispersion. A basal medium with heat resistant salt components of the minimal medium was given into the reactor before in-situ sterilisation (120 °C, 20 min). After cooling down to 37 °C, heat-sensitive components and glycerol were pumped aseptically into the bioreactor. For inoculation, 1.0 L of preculture were added via a pump from a sterile bottle. The initial reactor volume was 15.0 L. Throughout the process, pH was controlled by adding 25% NH_4_OH or 42% H_3_PO_4_ and maintained at a pH of 7.0. The dissolved oxygen concentration was kept above 30% air saturation by adjusting stirrer speed (200 min^−1^–1000 min^−1^), aeration rate (5 L min^−1^–40 L min^−1^) and pressure (up to 1.3 bar). Carbon dioxide production and oxygen uptake were monitored via an exhaust gas analyser (Easy Line, ABB Automation, Zurich Switzerland).

After the initial batch phase, two consecutive feeding solutions were supplied exponentially into the reactor: 1.0 L feeding solution A with (g L^−1^) 120.0 glycerol, 60.0 (NH_4_)_2_SO_4_, and 0.1 kanamycin, as well as 4.5 L feeding solution B with (g L^−1^) 400.0 glycerol, 25.0 (NH_4_)_2_SO_4_, and 0.1 kanamycin. The setpoint for exponential feeding was at a growth rate of µ_set_ = 0.1 h^−1^. Following the exponential feeding phases, 0.3 mM Isopropyl-β-D-thiogalactopyranosid (IPTG), as well as the initial amounts of MgSO_4_·H_2_O, CaCl_2_·2 H_2_O, FeSO_4_·7 H_2_O and thiamine, were aseptically injected into the reactor via syringes through a septum; a volumetrically constant feeding phase was initiated. Feed solution C (5.0 L) containing (g L^−1^) 800.0 glycerol, 8.0 (NH_4_)_2_SO_4_, 8.0 (NH_4_)_2_HPO_4_ and 0.1 kanamycin was applied with 0.2 g_glycerol_ g_CDW_^−1^, based on the biomass concentration at the end of the exponential feeding phase.

### Parallelised perturbation experiment for metabolic analysis

Metabolic analysis was conducted after rapid media transition [4] in parallel to the fed-batch production process with cells withdrawn through the bottom valve of the 42 L stirred-tank reactor, using the method of parallelised steady-state perturbation experiments adapted from [2]. A 1 L four-fold parallel stirred-tank lab-scale bioreactor system (DASGIP technology/Eppendorf, Jülich, Germany) was used with individual probes for online measurements of dissolved oxygen and pH. Each reactor was equipped with separate pumps for pH control with 21% H_3_PO_4_ and 2 M NaOH and feed supply, with a gas mixing unit for air, N_2_, and O_2_. Exhaust gas analyses were performed separately for each reactor.

While producing L-tryptophan at high rates during the constant feeding phase, 3.6 L cell suspension was taken from the fed-batch process on a 15 L scale. Following centrifugation (3260 g, 7.5 min, 37 °C; Rotixa centrifuge 50 RS, Hettich Zentrifugen, Tuttlingen, Germany), the supernatant was discarded, and cells were resuspended in 400 mL fresh minimal medium (37 °C) without carbon source. The cell suspension was distributed equally to the four parallel bioreactors, which contained 400 mL fresh minimal medium and 0.3 mM IPTG and 0.1% antifoam.

The parallel short-term perturbation studies lasted 21 min (pH 7, 37 °C, gassing 4 L min^−1^, stirrer speed 1200 min^−1^), and a three-stage feeding profile was applied with separate individual feeding solutions. All the feed supply rates were increased 9.0 and 15.0 min after inoculation. The first reactor was supplied with a feeding solution containing 100 g L^–1^ glycerol with 22 mL h^−1^, 44 mL h^−1^ and 66 mL h^−1^. In the feeding solution for the second reactor, glucose (80 g L^–1^) was used as a carbon source and was supplied with 24 mL h^−1^, 45 mL h^−1^ and 66 mL h^−1^. The feeding solution for the third reactor contained 100 g L^–1^glycerol and an additional 12 g L^–1^ shikimate; the supply rates were applied with 22 mL h^−1^, 44 mL h^−1^ and 66 mL h^−1^. Feeding solution 4 consisted of 80 g L^−1^ glucose and 9 g L^–1^ shikimate, the feeding rates for this reactor were 24 mL h^−1^, 45 mL h^−1^ and 66 mL h^−1^.

Samples for quantifying extracellular metabolites and determining cell dry weight concentrations were taken simultaneously from the reactors 1 min, 9 min, 15 min and 21 min after inoculation with a rapid sampling device, adapted from [54]. Samples were transferred to precooled centrifuge tubes containing glass beads for fast cooling. Samples for the quantification of intracellular metabolites were withdrawn from the parallel analysis reactors after 8 min, 14 min and 20 min and handled as described in Sect. 2.6. One sample for the quantification of intracellular metabolites was taken from the reference production process 10 min after initiating the metabolic analysis of the *E. coli* cells.

### Analytical methods

Cell dry weight of samples was determined in triplicates. In advance, 2 mL reaction tubes were dried (80 °C) and their empty weight was measured. Then, 2 mL of cell suspension was transferred to pre-weighed sample tubes and centrifuged (18,940 g, 4 °C, 20 min), the supernatant was discarded, and the reaction tube was dried and weighed again.

To determine extracellular metabolites by high-performance liquid chromatography (HPLC), samples were centrifuged (18,940 g, 4 °C, 10 min), and the supernatant was filtered (0.2 µm).

Amino acids were derivatised before analysis by an HPLC system (Smartline HPLC, Knauer, Berlin, Germany) with a fluorescence detector (RF20A detector, Shimadzu, Kyoto, Japan). Derivatisation was performed by the autosampler at 8 °C with o-phtaldialdehyde (OPA), mercaptopropionic acid (MCP) and iodoacetic acid (IAA), all in a 40 mM bicine buffer (pH 10.2). First, 10 µL of the sample was transferred (in 658 µL 0.3 mM MCP) to a separate destination vial. Then, 20 µL of IAA (3.5 mM) was added and mixed, followed by the addition of 70 µL OPA (11 mM in bicine/MeOH/MCP (928:7.1:0.1) (v/v/v)) and subsequent mixing. 20 µL of the derivatised sample dilution was injected into a Gemini column (C_18_ 150 × 4.6 mm, 5 µm; Phenomenex, Torrance, CA, USA), and the analytes were separated chromatographically at 40 °C for 43 min at a 1 mL min^−1^ flow rate. A gradient from solvent A (20 mM NaH_2_PO_4_, pH 7.6, filtered (0.2 µm)) and solvent B (MeOH/acetonitrile/water) (v/v/v)) was applied as follows: 0–3 min 100% solvent A, 8.5 min 75% solvent A, 30 min 60% solvent A, 30.02 min 0% solvent A, 32 min 0% solvent A, 32.02 20% solvent A, 34 min 20% solvent A, 38 min 100% solvent A, 43 min 100% solvent A.

Quantification of alcohols and organic acids was performed with an HPLC system (Agilent 1100 series, Agilent Technologies, Santa Clara, CA, USA) with a refractive index detector at 950 nm. A 20 µL volume of sample was used for injection; separation was conducted on an Aminex HPLX-87H column (BioRad, Munich, Germany) at 65 °C with 0.7 mL min^−1^ isocratic flow (5 mM H_2_SO_4_).

Glucose and ammonia were quantified enzymatically with assays from Boehringer Mannheim/R-Biopharm (Darmstadt, Germany; Kits No 10716251035 and 11,112,732,035).

### Rapid sampling and metabolome quantification

High-resolution sampling for the quantification of intracellular metabolites was performed using a rapid sampling device with inner tubes adapted from [54]. The sampling devices were filled with 22.5 mL of a quenching fluid [60% MeOH, 30 mM triethanolamine (TEA)], and then they were hermetically sealed and cooled down to − 60 °C in a cryostat. Immediately before sampling, the internal pressure was reduced to 0.85 bar, and the sampling device was then coupled to the sample point in the bioreactor. Due to the vacuum generated, the sample volume was sucked into the sampling device and distributed in the quenching fluid through small cavities within the inner tubes to achieve immediate inactivation of the cells’ metabolism. The inactivated cell mixture was transferred quickly into precooled centrifuge tubes (− 40 °C ethanol/ice-mixture). After mixing, 1 mL of the mixture was extracted in 2 mL of TEA buffer (30 mM, pH 7) for 5 min. The extraction was conducted in triplicate; in two extraction mixtures, 350 µL of U-^13^C cell extract as internal standard (undiluted and diluted 1:10, respectively) was added. The U-13C cell extract was prepared as described by [55] with NT1259 *shiA* pF*aroFBL*_Kan_. After extraction, the samples were cooled and centrifuged (20 min, 3260 g), and the supernatant was stored at − 80 °C until analysed by mass spectrometric analysis (TSQ Vantage, Thermo Fisher Scientific, Waltham; MA, USA). For analysis, a UHPLC-MS/MS method adapted from Buescher [56] was used. 20 µL of the sample was injected on an Acquidity HSS T3 column (150 mm × 2.1 mm × 1.8 µm; Waters Corporation, Milford, USA), and analytes were separated chromatographically at 40 °C for 36 min. A gradient of solvent A (10 mM tributylamine, 15 mM acetic acid, 5% (v/v) MeOH) and solvent B (100% isopropanol) was applied [56]. Solvents used were of ultra-pure MS quality. Ionisation within the detector was generated with 2.0 kV spray voltage and 400 °C vaporiser temperature. Sheath gas pressure was set to 5.0, and aux gas pressure to 20.0 (arbitrary units); then the capillary was heated to 380 °C.

### RT-qPCR analysis

Total RNA for the RT-qPCR was prepared using a Quick RNA mini kit (Qiagen, Hilden, Germany). The RNA quality was analysed by running RNA-agarose gel electrophoresis. The cDNA was created using EpiScript Reverse Transcriptase (Biozym, Hessisch Oldendorf, Germany) with a 6 N random primer. The real-time quantitative PCR (RT-qPCR) was carried out using Blue S-Green qPCR kit (Biozym, Germany) according to the supplier’s recommendations, using the ∆∆Ct comparative method. The primers used for this analysis are listed in Table 1. The *ftsZ* gene was used as endogenous calibrator for the experiment. The run was performed in a qTower system (Analytik Jena, Jena, Germany). Data were analysed using the qPCRsoft 3.4 software application (Analytik Jena).

### Thermodynamics-based analysis of intracellular flux distributions

Thermodynamics-based flux variability analysis (TFA) was performed with a python package implemented by Salvy [14], which integrates thermodynamic information into genome-scale metabolic models and allows constraint-base modelling, considering measured intracellular metabolite concentrations [14]. The model used, was *i*JO1366 [57]. Genome-wide models are represented as a complex system of linear equations in form of a stoichiometric matrix N with n reactions and m metabolites. When steady-state conditions are fulfilled, the linear system of equotations can be solved through the maximisation of a chosen objective function. In this work, growth was chosen as target function and to restrict the solution space, extracellular rates (uptake of substrates, production of L-trp and by-product formation as well as respiration) were used, which have been measured during the perturbation experiments and the reference state of the L-tryptophan production process. Extracellular pH was set to pH 7.0 and the ionic strength to 0.15 M to adapt thermodynamic calculations to in vivo conditions. Measured intracellular metabolite concentrations were also implied to eliminate flux distributions that contradict thermodynamic laws. Thermodynamic information on metabolites was already included in the model [14] or added from the Equilibrator database [9]. The computations enabled the determination of alternative metabolic routes that fulfil 99.9% of the optimal solution and the estimation of flux ranges for each reaction in the model and feasible ranges for Gibbs energies of reactions and metabolite concentrations. The solution space was further examined by a sampling function (OptGpSampler [58]) with 10,000 computing cycles.

### Metabolic control analysis

The calculations for MCA were based on the linearisation approach by Visser and Heijnen [59] and were carried out using MATLAB 2016b (Mathworks, Natick, MA, USA). The principle of the MCA theorem is the linearisation of mass balance equations, referred to a reference steady-state. In this work, the L-tryptophan production process served as a reference state for the 12 steady states during metabolic analysis. The prerequisite for MCA is the estimation of elasticities $\varepsilon_{{X_{i} }}^{{v_{j} }}$. As local parameters, elasticities mathematically describe the change in reaction rate *v*_*j*_ upon the change in metabolite concentration level *X*_*i*_ and are defined as (Eq. 1):

1 $$\varepsilon_{{X_{i} }}^{{v_{j} }} \equiv \frac{{X_{i} }}{{v_{j} }}\frac{{\partial v_{j} }}{{\partial X_{i} }} = \frac{{\partial \ln v_{j} }}{{\partial \ln X_{i} }}$$

Negative elasticities express inhibitions, whereas positive values describe activating effects.

For reactions operating close to thermodynamic equilibrium, the estimation of elasticities was achieved by a thermokinetic approach [60] (Eq. 2), whereby the stoichiometric coefficient *n*_*ij*_ represents the moles of metabolite *I* consumed or formed in reaction *j* while *A* describes the thermodynamic driving forces, depending on the reaction temperature *T* and universal gas constant *R.*

2 $$\varepsilon_{{X_{i} }}^{{v_{j} }} = \frac{{ - n_{ij} R \cdot T}}{{A_{j} - A_{j}^{\# } }}$$

For reactions near to the thermodynamic equilibrium, $A_{j}^{\# }$ equals zero, and the elasticity coefficient can be directly derived from the reaction affinity, described by the negative Gibbs free energy of reaction.

For reactions operating far from equilibrium, an active regulation is assumed, and therefore effectors $e$ in the form of regulating metabolites were defined. Information on regulatory relations was taken from the literature. With the following lin-log approach [61] (Eq. 3), the coefficients were estimated by performing Monte Carlo sampling of flux ranges and concentration ranges.

3 $$\left[ {\frac{v}{{v^{0} }}\frac{{e^{0} }}{e}} \right] - 1 = \left[ {\ln \frac{X}{{X_{0} }}} \right]\left[ {\varepsilon_{X}^{v} } \right]$$

The elasticities obtained were summarised in matrix ${\text{E}}_{{{\text{X}}_{0} }}$ ${E}_{{X}_{0}}$ and enabled the estimation of flux control coefficients *C* $C$ using the MCA linearisation approach [59], which is described by (Eq. 4) with stoichiometric matrix $N$ *N*, reduced stoichiometric matrix ${N}_{R}$ *N*_*R*_ Link-Matrix ${L}_{0}$ *L*_*0*_ and independent metabolites ${X}^{ind}$ *X*^*ind*^ Indexing with 0 refers to the reference state, described by the fluxes, enzymes and metabolite concentrations of cells from the L-tryptophan production process at the time of metabolic analysis.

$$C_{{e_{j} }}^{{v_{i} }} = \frac{{e_{j} }}{{v_{i} }}\frac{{dv_{i} }}{{de_{j} }} = E_{{X_{0} }} \left\{ { - L_{0} \cdot \left( {N_{R} \times \left[ {v_{0} } \right] \times E_{{X_{0} }} \times L_{0} } \right)^{ - 1} \cdot N_{R} \cdot \left[ {v_{0} } \right]} \right\}$$

with

4 $$L_{0} = \left[ {X_{0} } \right]^{ - 1} \cdot N\;\left( {N_{R} } \right)^{\# } \cdot \left[ {X_{0}^{ind} } \right]$$

Flux control coefficients represent the effects of a 1% increase of enzymatic activity on a specific metabolic flux in the model. In this work, a reduced metabolic model (50 reactions and 57 metabolites) including glycolysis, glycerol metabolism, tricarboxylic acid (TCA) cycle, pentose-phosphate-pathway (PPP), L-serine biosynthesis and biosynthesis of aromatic amino acids, was used. Uncertainties of estimated flux distributions, Gibbs free energies of reaction and metabolite concentrations were taken into consideration through Monte Carlo sampling (10,000 cycles) [62].

## Results and discussion

### L-tryptophan production from glycerol on a 15 L scale

**Fig. 2:**
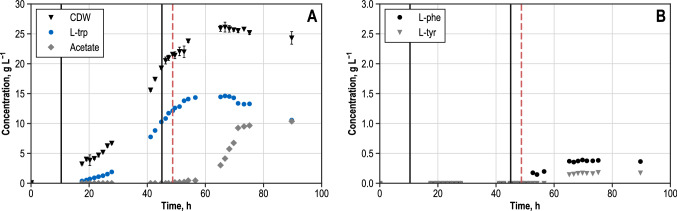
Fed-batch production of L-tryptophan with *E. coli* NT1259 *shiACg* pF112*aroFBL*_Kan_ on a 15 L scale (37 °C, pH 7.0, DO > 30% air saturation). A: Concentrations (unit: g L^−1^) of cell dry weight (CDW), L-tryptophan (L-trp) and acetic acid. B: Concentrations of L-phenylalanine (L-phe) and L-tyrosine (L-tyr). Vertical solid black lines indicate (i) the end of the batch phase (10.4 h) and (ii) the beginning of the constant feeding phase/addition of IPTG (45.1 h). The broken line marks the process time for cell sampling for the parallel metabolic perturbation studies (48.8 h)

This work is intended to study the metabolic control of L-tryptophan production in *E. coli cells* with glycerol as a carbon source, focusing on fluxes within the biosynthesis of aromatic amino acids. In a first step, the successful integration and expression of the *shiA*-transporter gene was verified by RT-qPCR (Supplementary Fig. 1) and the shikimate uptake by L-tryptophan producing cells without shikimate-transporter (*E. coli* NT1259 pF112*aroFB*L_Kan_ was analysed in comparison to cells with the integrated shikimate-transporter (*E. coli* NT1259 *shiACg* pF112*aroFB*L_Kan_) in parallel cultivations in shake-flasks. The integration of the *shiA*-gene from *Corynebacterium glutamicum* enabled the uptake of shikimate with a measured rate of 0.26 mmol g_CDW_^−1^ h^−1^ in shake-flask, whereas the concentration of shikimate remained constant during the cultivation of the cells without transporter (Supplementary Fig. 2). The underlying reference fed-batch process for the cultivation of the shikimate-transporter strain was established before [37]. This fed-batch process has been applied with the *E. coli* L-tryptophan producer [17], used in this work but without the additional shikimate transporter. The identical production process described in [17, 37] was used to cultivate *E. coli* NT1259 *shiACg* pF112*aroFB*L_Kan_ in this work. Process performance on a 15 L scale of *E. coli* NT1259 *shiACg* pF112*aroFB*L_Kan_ is summarised in Fig. 2 with concentrations of cell dry weight, L-tryptophan and acetate (Fig. 2A) and the by-products L-tyrosine and L-phenylalanine (Fig. 2B) as a function of process time. When the initial batch phase had ended, an exponential glycerol feeding was applied, resulting in a cell dry weight concentration of 17.4 g L^−1^ after 42.7 h (*µ* = 0.07 h^−1^) (Fig. 2A). At 66.7 h, the cell dry weight concentration of 26.1 g L^−1^ was reached, slightly decreasing to 24.3 g L^−1^ by the end of the process (89.7 h). The L-tryptophan concentration steadily increased during exponential feeding, leading to a product concentration of 8.8 g L^−1^ L-tryptophan at the end of the first process phase. At a process time of 45.1 h, exponential feeding was switched to constant feeding with a glycerol feeding rate of 0.2 g_gly_ g_CDW_^−1^ h^−1^ and 0.3 mM IPTG were added for the induction of gene expressions. L-tryptophan concentration further increased to 14.6 g L^−1^ until 66.8 h. The highest cell-specific production rate of 23 mg_L-trp_ g_CDW_^−1^ h^−1^ was determined at a process time of 56.6 h. After 66.8 h no further product formation was observed. Due to dilution by feeding and base addition, the product concentration declined to 10.6 g L^−1^ until the end of the process.

During the batch phase, 4 g L^−1^ of the initial glycerol was consumed. No glycerol accumulation was observed until 52.7 h. Afterwards, glycerol concentration in the medium started to rise so that a maximum concentration of 73.0 g L^−1^ was observed at the end of the fed-batch process (data not shown). Throughout the process, ammonia was present in concentrations between 2–4 g L^−1^. After a process time of 65 h, ammonia accumulated to 7.1 g L^−1^ at the end of the process (data not shown) due to reduced uptake by the cells. By-product formation of L-phenylalanine and L-tyrosine started after induction, and the aromatic amino acids accumulated to 0.4 g L^−1^ L-phenylalanine and 0.2 g L^−1^ L-tyrosine. Acetate production was initiated 49.5 h after inoculation; the maximum acetate concentration of 10.4 g L^−1^ was reached at the end of the process. Additionally, 1.0 g L^−1^ lactate and 0.8 g L^−1^ succinate was present at the end of the process (data not shown).

In comparing the process performance data of *E. coli* NT1259 *shiA* pF112*aroFB*L_Kan_ with *E. coli* NT1259 pF112*aroFB*L_Kan_ [17], it emerges that there are no significant deviations in the main state variables. The differences are within the observation variance. The maximum product concentration with *E. coli* NT1259 pF112*aroFB*L_Kan_ was 14.3 g L^−1^, the maximum production rate after induction was also 23 mg_L-trp_ g_CDW_^−1^ h^−1^ and the maximum cell dry weight concentration (31.8 g L^−1^) was slightly higher compared to *E. coli* NT1259 *shiA* pF112*aroFB*L_Kan_ (26.1 g L^−1^). It can thus be deduced that the insertion and expression of the gene encoding an additional shikimate transporter does not influence the process performance significantly.

### Metabolic analysis during L-tryptophan production using shikimate as effector

L-tryptophan production of the fed-batch process shown above was further examined with parallelised short-term perturbation experiments, which were conducted locally separated from the reference process. 3.6 L of cell suspension was sampled 4 h after induction during the constant feeding phase at a process time of 48.8 h. High cell-specific L-tryptophan production rates were observed at this timepoint, and the cell dry weight concentration was 22.4 g L^−1^.

The metabolic analysis is based on the perturbation of the cells’ metabolism, caused by a change in substrates and substrate supply rates in short-term studies. To achieve an intense deflection within the L-tryptophan biosynthesis pathway, shikimate was used as an additional effector substrate, supplementing the main carbon sources glycerol and glucose applied for the perturbation experiments. Rapid sampling enabled the quantification of the cells’ reactions to the varying substrate supplies by evaluating the extra- and intracellular metabolome. The proteome is assumed to be unchanged for the total analysis time of 21 min [2, 4]. Data from the four analysis reactors were evaluated and referred to the reference process during metabolic analysis. In the first reactor, glycerol was used as substrate; in the second reactor glucose was supplied as the sole carbon source. In the third reactor, glycerol and shikimate were fed in, and in the fourth reactor glucose and shikimate were supplied. A three-stage constant and limiting feeding profile was applied in all the reactors, which led to 13 metabolic steady states in total, including the reference state.

#### Extracellular fluxome

**Fig. 3:**
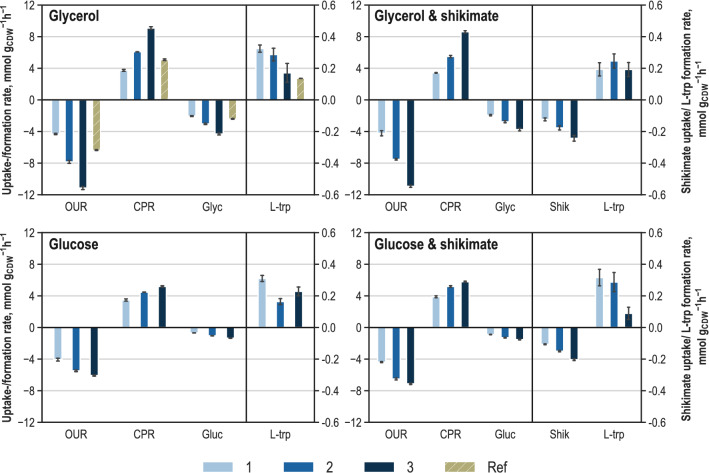
Extracellular uptake and formation rates (unit: mmol g_CDW_^−1^ h^−1^) of substrates and products and respiration during parallelised short-term perturbation experiments in stirred-tank reactors for the metabolic analysis of *E. coli* NT1259 *shiA*_*Cg*_ pF112*aroFBL*_Kan_ producing L-tryptophan with glycerol, glycerol and shikimate, glucose, and glucose and shikimate as perturbation substrates. Respiration rates: oxygen uptake (OUR) and CO_2_ production (CPR); biomass specific substrate uptake rates of glycerol (Glyc), glucose (Gluc) and shikimate (Shik) and biomass specific production rates of L-tryptophan (L-trp) are depicted. 1–3: metabolic steady-state conditions, resulting from a corresponding three-stage feeding profile. Ref: Extracellular rates in the 15 L fed-batch reference process during metabolic analysis time

Samples for quantifying extracellular metabolites were taken at the beginning and end of each substrate supply level. Figure 3 summarises the biomass specific rates for substrate uptake, product formation and respiration during the metabolic analysis of the 13 metabolic steady states. Biomass specific rates from the reference process during metabolic analysis are included in the graph of the metabolic analysis with glycerol as the sole carbon source. For respiration rates, the mean value during the corresponding metabolic steady-state is depicted (online sampling with an interval of 5 s).

In all analysis reactors, the three-step feeding profile was clearly reflected by the substrate uptake and respiration rates. With glycerol as perturbation substrate, the substrate uptake was increased from 2.0 mmol g_CDW_^−1^ h^−1^ in the first feeding stage to 4.3 mmol g_CDW_^−1^ h^−1^ at the highest feed rate. This led to three corresponding differential gradations in oxygen uptake and carbon dioxide production rates. Product formation was not increased with elevated substrate uptake rates. During the first feeding stage, a high L-tryptophan production rate of 0.32 mmol g_CDW_^−1^ h^−1^ was reached, whereas the lowest L-tryptophan production rate of 0.17 mmol g_CDW_^−1^ h^−1^ was measured during the last feeding period. During the second and third substrate supply level, acetate started to accumulate at an increasing rate (data not shown). Substrate uptake and respiration rates from the reference process during metabolic analysis lay between the lowest and intermediate perturbation steady-state rates. From this result, it can be deduced that the deflection in the metabolism of L-tryptophan producing cells from the reference process was successful in both directions.

In the analysis reactor with glycerol and supplementary shikimate as carbon sources, the lowest uptake rate of glycerol was 1.9 mmol g_CDW_^−1^ h^−1^ and 0.12 mmol shikimate g_CDW_^−1^ h^−1^. The uptake rates increased in two equal steps to 3.8 mmol glycerol g_CDW_^−1^ h^−1^ and 0.24 mmol shikimate g_CDW_^−1^ h^−1^ during the last supply level. Respiration rates were staggered, corresponding to the substrate uptake rates. Oxygen uptake and carbon dioxide production were slightly lower than the analysis reactor with glycerol as the sole carbon source. Constant L-tryptophan production rates of around 0.2 mmol g_CDW_^−1^ h^−1^ were determined within the estimation error. Thus, an increase in substrate supply rate did not result in varying product formation rates. In contrast, the by-products acetate and succinate accumulated with increasing rates during the second and third supply level (data not shown).

With glucose as the carbon source, the substrate uptake rate was increased from 0.7 mmol g_CDW_^−1^ h^−1^ at the lowest supply level to 1.4 mmol g_CDW_^−1^ h^−1^ at the highest. The response of the *E. coli* cells to glucose supply became apparent in three stages of respiration rates, whereas the differences were less between the second and third supply levels than between the first and second. The product formation rate was 0.31 mmol L-tryptophan g_CDW_^−1^ h^−1^ during the first feeding stage and decreased to 0.16 mmol L-tryptophan g_CDW_^−1^ h^−1^ during the second and rose again to 0.23 mmol L-tryptophan g_CDW_^−1^ h^−1^ during the last. Small amounts of acetate, ethanol and succinate were formed during the last feeding stage (data not shown).

In the analysis reactor where glucose and shikimate were feeded in, the glucose uptake rate increased from 0.9 mmol g_CDW_^−1^ h^−1^ during the first feeding period to 1.5 mmol g_CDW_^−1^ h^−1^ during the last. The shikimate uptake rate rose from 0.11 mmol g_CDW_^−1^ h^−1^ during the lowest perturbation steady state to 0.2 mmol g_CDW_^−1^ h^−1^ during the highest. Respiration rates were similar to the analysis with glucose as the sole carbon source, although total values were slightly higher. L-tryptophan formation rate was constant at 0.27 mmol g_CDW_^−1^ h^−1^ during the first and second supply level and decreased to a very low rate of 0.09 mmol g_CDW_^−1^ h^−1^ during the last. During the last feeding stage, acetate and formate were formed, ethanol production occurred throughout the analysis time with increasing rates (data not shown). The dissolved oxygen concentration decreased to 10% air saturation during the last feeding stage when glycerol was used as the perturbation substrate.

#### Intracellular metabolome

**Fig. 4:**
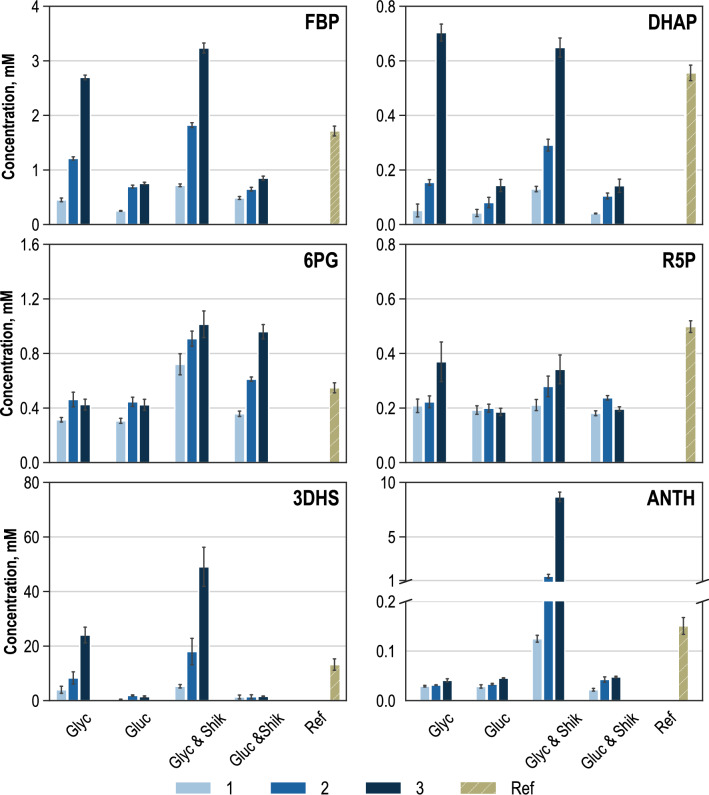
Intracellular concentrations (unit: mM) of fructose 1,6-bisphosphate (FBP), dihydroxyacetone-phosphate (DHAP), 6-phosphogluconate (6PG), ribose-5-phosphate (R5P), 3-dehydroshikimate (3DHS), anthranilate (ANTH) during parallelised short-term perturbation experiments in stirred-tank bioreactors with the carbon sources glycerol (Glyc), glycerol and additional shikimate (Glyc & Shik), glucose (Gluc) and glucose with additional shikimate (Gluc & Shik). 1–3: steady-state conditions corresponding to three-stage feeding levels. Ref: Intracellular concentrations in the reference fed-batch process during metabolic analysis

Rapid sampling for metabolome quantification took place at the end of each substrate supply phase, simultaneously from the four analysis reactors and the reference fed-batch production process during metabolic analysis. Aside from extracellular fluxome data, the examination of intracellular metabolite concentrations provides insight into the metabolic state of the cells and enables the quantification of the cells’ reaction to the perturbation. In total, 45 intracellular metabolites of relevance for the production of L-tryptophan were quantified from glycolysis, glycerol metabolism, TCA cycle, PPP, L-serine biosynthesis and synthesis of aromatic amino acids. A representative variety of metabolites is depicted in Fig. 4, which clearly reflects the disturbance of the metabolism around the reference state in the 15 L fed-batch production process. The deviations in intracellular metabolite concentrations are dependent on the substrates and the applied substrate supply rates. The metabolites shown exemplarily are fructose-1,6-bisphosphate (FBP), dihydroxyacetone-phosphate (DHAP), 6-phosphogluconate (6PG), ribose-5-phosphate (R5P), 3-dehydroshikimate (3DHS) and anthranilate (ANTH).

FBP participates as a central metabolite in glycolysis and gluconeogenesis and is formed either from fructose-6-phosphate in glycolytic direction or from DHAP and glyceraldehyde 3-phosphate in gluconeogenetic direction. For FBP, rising intracellular concentrations were measured with all carbon sources. This effect is more pronounced, when glycerol is used as main carbon source instead of glucose and is further enhanced when shikimate is used as additional substrate. The intracellular concentrations of FBP varied from 0.45 mM to 3.23 mM. The intracellular FBP concentration of the cells of the sample from the reference process was 1.7 mM and fits well between steady-state 2 and 3 of the glycerol perturbation experiment.

DHAP is formed at the connection point of glycolysis and gluconeogenesis and rising intracellular concentrations are an immediate consequence of the increasing glycerol and glucose supply during metabolic analysis. The increase is significantly more distinct when glycerol was used as carbon source. Intracellular DHAP concentrations ranged from 0.04 mM to 0.70 mM. The measured concentration in the reference state sample was comparable to the concentrations reached with glycerol as perturbation substrate.

6PG and R5P were chosen as representative metabolites from PPP. The 6PG is located in the oxidative part of the PPP and R5P acts as immediate precursor metabolite for 5-phospho-alpha-D-ribose 1-diphosphate (PRPP), an important precursor in L-tryptophan biosynthesis. Intracellular 6PG concentrations were almost constant at around 0.4 mM in the perturbation experiments with glycerol and glucose as carbon sources. An increase in concentrations was measured in both cases when shikimate was used as secondary perturbation substrate. The highest intracellular concentration of 1.01 mM 6PG was reached during the third steady-state with glycerol and shikimate as perturbation substrates. The intracellular 6PG concentration in the sample from the reference process was slightly higher than in the perturbation experiment with glycerol as sole carbon source. However, it was lower than the concentration measured during the first steady state with glycerol and additional shikimate. Changes observed in intracellular R5P concentrations were less significant. Rising intracellular concentrations from 0.22 mM to 0.33 mM were measured for the two analyses with glycerol as carbon source and constant values of around 0.20 mM R5P were obtained for both analyses with glucose as carbon source. The intracellular concentration measured in the cells of the sample from the reference state marginally exceeded the intracellular concentration of steady-state 3 with glycerol as perturbation substrate.

As part of the chorismate biosynthesis, 3DHS is formed from 3-dehydroquinate and is the direct precursor of shikimate. Whereas the intracellular concentrations stay below 2.00 mM when glucose is used as carbon source, intracellular 3DHS concentrations rose to 24.04 mM in the third steady state when glycerol was used as sole carbon source and even higher to 51.89 mM when shikimate was added to glycerol. This observation has been described earlier [2, 17]. The intensified increase in intracellular 3DHS concentrations with shikimate as an additional substrate supplementing glycerol can be explained by the reversibility of shikimate dehydrogenase, which catalyses the conversion of 3DHS to shikimate and vice versa [63]. Since 3-dehydroquinate (3DHQ) cannot be measured with the LC–MS method used in this work, a back flux originating from shikimate to 3DHQ cannot be determined.

A good accordance was obtained between the intracellular 3DHS concentration of the reference process and the metabolic analyses experiment with glycerol as sole carbon source.

ANTH is the first metabolite downstream from chorismate in the direction of L-tryptophan biosynthesis and ANTH is combined with PRPP by the anthranilate synthase, whereby N-(5-phosphoribosyl)-anthranilate (PRAN) is formed (Fig. 1). Whereas the intracellular concentrations remained below 0.05 mM ANTH after perturbation with glycerol as sole carbon source and both perturbations with glucose, the intracellular ANTH concentrations in the metabolic analysis with glycerol and additional shikimate as substrates strongly increased from 0.02 mM in the first to 8.76 mM in the third steady state. This noticeable finding may indicate limitations in anthranilate conversion, either caused by low enzymatic capacity of the anthranilate synthase or insufficient precursor supply by the PPP.

These datasets reveal clear deflections throughout the central carbon metabolism of *E. coli*, induced by the supply of different substrates and changes in substrate supply rates. Shikimate addition triggered strong deviations, especially in the biosynthesis pathway of chorismate and in L-tryptophan biosynthesis, which become visible in changes in intracellular metabolite concentrations. However, the influence of extracellular shikimate supply is not constrained to the biosynthesis of aromatic amino acids; it is also detectable in intracellular metabolite concentrations in the PPP and the glycolysis.

### Thermodynamics-based analysis of the metabolic network

Extracellular fluxome and intracellular metabolite concentrations provide a large data volume, which allows quantifying the cells’ metabolic state and capacity during the different metabolic steady states achieved in the perturbation experiments. In general, measured extracellular rates enable the theoretic estimation of intracellular flux distributions within a genome-wide metabolic model with methods like flux balance and flux variability analysis. These computations were improved by taking thermodynamic constraints of the examined metabolic network into account, using thermodynamics-based flux analysis (TFA) [12, 14]. Thus, flux distributions, which contradict cell physiology and thermodynamic laws, were excluded. Moreover, quantitative metabolomics were included in the model to estimate their effects on the direction of metabolic reactions [12, 14]. Thermodynamic analyses of the metabolic network allow estimations of the displacement from thermodynamic equilibrium of each reaction in the model, as well as concentration ranges of non-measurable metabolites. The genome-wide *E. coli* model used in this work was *i*JO1366, containing 2,583 reactions and 1805 metabolites [57]. Maximisation of biomass formation was chosen as an objective function, and the measured extracellular rates and metabolite concentrations were used to constrain the solution space.

### Thermodynamics-based flux estimations

**Fig. 5:**
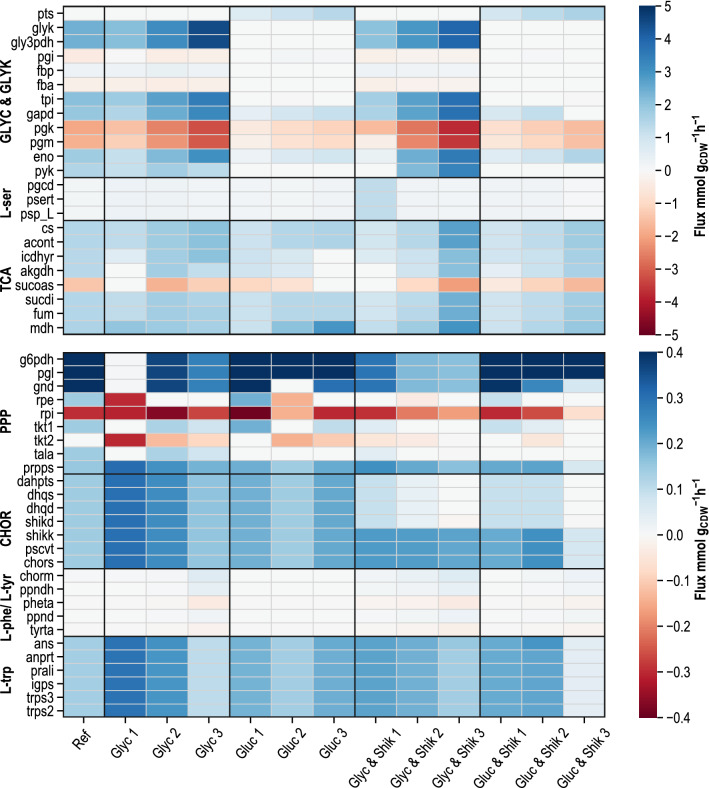
Heat map illustrating flux distributions (unit: mmol g_CDW_^−1^ h^−1^) in glycolysis and glycerol metabolism (GLYC & GLYK), TCA cycle (TCA), pentose-phosphate-pathway (PPP), L-serine biosynthesis (L-ser), chorismate biosynthesis (CHOR), L-phenylalanine and L-tyrosine biosynthesis (L-phe/L-tyr) and L-tryptophan production (L-trp) derived by thermodynamics-based flux analysis (pyTFA), restricted by measured extracellular rates during the parallel short-term perturbation experiments in stirred-tank bioreactors for metabolic analysis of L-tryptophan producing *E. coli* cells. Fluxes are depicted for the reference L-tryptophan production process with glycerol as sole carbon source (Ref) and the analysis reactors with glycerol (Glyc), glucose (Gluc), glycerol and shikimate (Glyc & Shik) as well as glucose and shikimate (Gluc & Shik). Flux directions are defined as in the model *i*JO1366

Figure 5 summarises the estimated flux distributions of reactions with relevance for the L-tryptophan biosynthesis. The upper heatmap shows reactions from glycerol metabolism and glycolysis (GLYC & GLYK), L-serine biosynthesis (L-ser) and citric acid (TCA) cycle with flux values ranging from − 5 mmol g_CDW_^−1^ h^−1^ to 5 mmol g_CDW_^−1^ h^−1^. The lower heatmap comprises reactions of the PPP, chorismate biosynthesis (CHOR), by-product formation of L-phenylalanine and L-tyrosine (L-phe/L-tyr), as well as L-tryptophan biosynthesis (L-trp) with low rates from − 0.4 mmol g_CDW_^−1^ h^−1^ to 0.4 mmol g_CDW_^−1^ h^−1^.

Activities of PTS and glycerol degradation (glyk and gly3pdh) are predetermined by the primary carbon source, which was used for perturbation. Fluxes through PTS increase with higher glucose supply rates and are inactive when glycerol is used as the primary carbon source. With increasing glycerol uptake rates, fluxes in aerobic glycerol metabolism are enhanced. In the upper part of glycolysis, reactions between glucose-6-phosphate and DHAP are inverted into the gluconeogenetic direction, when glycerol is used as carbon source. Glycolytic fluxes downstream from glyceraldehyde 3-phosphate are estimated to be higher when glycerol is used as the primary substrate than with the steady states where glucose was consumed. Pyruvate kinase was only active at glycerol consumption. In cases where glucose was used as the primary substrate, phosphoenolpyruvate conversion is executed by PTS when glucose is taken up and at the same time converted into glucose-6-phosphate.

Fluxes within L-serine biosynthesis vary from 0.21 mmol g_CDW_^−1^ h^−1^ to 0.91 mmol g_CDW_^−1^ h^−1^, and their estimated activity is mainly influenced by measured product biosynthesis, biomass formation and L-serine usage in other parts of the metabolism.

The metabolic reactions point in the same direction for all substrate combinations in the TCA cycle that were used for metabolic analysis. The computed activities within the TCA cycle rise with the increase of substrate uptake for each of the datasets. Good accordance was obtained between the reference state and the perturbation experiment with glycerol for reactions of glycolysis, glycerol metabolism, L-serine biosynthesis and the TCA cycle due to the high similarity in the measured (and therefore predetermined) uptake and production rates.

Flux distributions within the PPP are afflicted with uncertainties due to redundancies and reversibility of several reactions. However, through restriction with measured rates and intracellular metabolite concentrations, varying flux directions are determined only for the reactions of transketolase (tkt1 and tkt2), ribulose 5-phosphate 3-epimerase (rpe), and transaldolase (tala). Activities are estimated for the oxidative and the non-oxidative pathway of the PPP. Fluxes through PRPP synthetase (prpps) are mainly predetermined by the measured and prescribed L-tryptophan production rate.

Likewise, the flux distributions in the aromatic amino acid biosynthesis are defined through the measured L-tryptophan-, L-phenylalanine and L-tyrosine production rates, which served as constraints for the estimations.

In chorismate biosynthesis, strong deviations appear due to the supplementary feeding of shikimate in two reactors. Fluxes in L-phenylalanine and L-tyrosine production remain below 0.04 mmol g_CDW_^−1^ h^−1^. In L-tryptophan biosynthesis, deviant fluxes were measured for the different steady states. However, an increase in substrate uptake rates did not lead to enhanced fluxes within L-tryptophan biosynthesis. Hypothetically assumed, that all the supplemented shikimate could be channelled to chorismate on top of the already synthesised chorismate from erythrose-4-phosphate and phosphoenolpyruvate, the product formation is estimated to be increased by 108% at the maximum. Comparing the results of the metabolic analysis with glycerol and of cells from the reference process, a high comparability between the two steady states was also found for the reactions of the PPP and the biosynthesis of aromatic amino acids.

The estimated intracellular flux distributions reveal clear deflections throughout the metabolism originating from changes in substrate supply. Shikimate addition led to significant deviations, especially in chorismate biosynthesis. In all, 13 steady states were characterised by thermodynamics-based flux analysis, which was performed using the experimentally acquired intracellular and extracellular metabolome data.

### Estimation of Gibbs energies of reactions

**Fig. 6:**
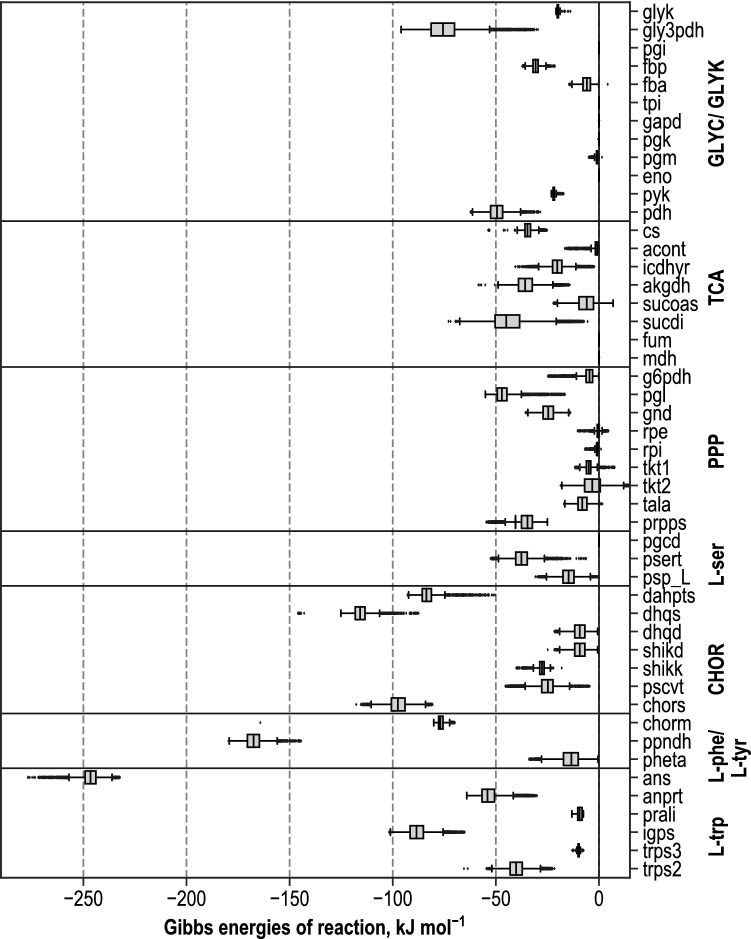
Estimated Gibbs energies of reactions (unit: kJ mol^−1^) for reactions of glycolysis and glycerol metabolism (GLYC & GLYK), TCA cycle (TCA), pentose-phosphate-pathway (PPP), L-serine biosynthesis (L-ser), chorismate biosynthesis (CHOR), L-phenylalanine and L-tyrosine biosynthesis (L-phe/L-tyr) and L-tryptophan production (L-trp) of cells in the L-tryptophan fed-batch production process at the process time chosen for metabolic analysis, derived by thermodynamic flux analysis (pyTFA) in consideration of measured intracellular metabolite concentrations and Gibbs’ free energies of formation for intracellular pH of pH 7.5 and ionic strength of 0.15 M. Reaction energies are depicted for positive flux directions, estimated with thermodynamics-based flux analysis for the reference state

With pyTFA thermodynamic feasibility studies are directly coupled to flux variability analysis in the same computation approach. As a result, flux distributions are received, as well as thermodynamic profiles of the analysed steady states. The displacement from the thermodynamic equilibrium of reactions is defined by the Gibbs energies of reactions, which is crucial information for MCA. Reactions operating close to thermodynamic equilibrium can be distinguished by Gibbs energies close to zero, whereas reactions that operate far from equilibrium have Gibbs energies with an offset from zero of at least −10 kJ mol^−1^ [8]. The feasibility of the model is dependent on negative Gibbs reaction energies of all active fluxes. Reaction directions are not constrained. Thus positive Gibbs energies may also occur for some reversible reactions. Feasible ranges of Gibbs energies of reactions were estimated for the steady state in the reference production process during the metabolic analysis when high production rates were achieved. The range of values was further examined via a sampling function with 10,000 sampling points taken from flux and metabolite concentration ranges [58]. Gibbs reaction energies for the considered pathways are depicted in Fig. 6.

Gibbs energies close to zero were obtained in glycolysis and glycerol metabolism for the reactions glucose-6-phosphate isomerase (pgi), fructose-bisphosphate aldolase (fba), triose-phosphate isomerase (tpi), glyceraldehyde-3-phosphate dehydrogenase (gapd), phosphoglycerate kinase (pgk), phosphoglycerate mutase (pgm) and enolase (eno), similar results were published by Kümmel et al. [64]. Highly negative reaction energies from − 24 kJ mol^−1^ to − 5 kJ mol^−1^ were calculated for glycerol-3-phosphate dehydrogenase (gly3pdh). Canelas [65] estimated Gibbs energies between − 16 kJ mol^−1^ and − 14 kJ mol^−1^ for this enzyme. Glycerol kinase (glyk), fructose-1,6-bisphosphatase (fbp), pyruvate kinase (pyk) and pyruvate dehydrogenase (pdh) were classified as operating far from equilibrium, according to their highly negative Gibbs reaction energies. In the TCA cycle, only succinyl-CoA synthetase (sucoas) showed an invertible direction of the reaction. For the enzymes aconitase A and B (acont), fumarase (fum) and malate dehydrogenase (mdh), Gibbs reactions energies were estimated to be close to zero. Gibbs reaction energies close to zero were received in good accordance with the literature [64, 65] in the PPP for the reactions of tkt1, tkt2, tala, rpe and ribose-5-phosphate isomerase (rpi), whereas the metabolic reactions of tkt1, tkt2, tala and rpe were found to be reversible.

Reactions with Δ_r_G’ close to zero are not likely to be subject to active regulation as their responsiveness to minimal changes in their reactants is very sensitive [11]. Phosphoglycerate dehydrogenase catalyses the first step of L-serine biosynthesis, starting from 3-phospho-D-glycerate (3PG). The estimates in this work indicate an operation close to the thermodynamic equilibrium of this reaction, which has also been described by Henry et al. [11]. Gibbs energies of < -50 kJ mol^−1^ were obtained for other reactions of the L-serine biosynthesis. Highly negative values for Δ_r_G’ were calculated for several reactions in the aromatic amino acid biosynthesis, indicating a strong metabolic regulation of these enzymes. Exceptional negative values were estimated for 3-deoxy -7-phosphoheptulonate synthase (dahpts), 3-dehydroquinate synthase (dhqs), chorismate synthase (chors), chorismate mutase (chorm), prephenate dehydratase (ppndh), anthranilate synthase (ans), anthranilate phosphoribosyltransferase (anprt), indole-3-glycerol-phosphate synthase (igps) and tryptophan synthase (trps2). Likewise, Henry [11] suggested strong regulations of dahpts, dhqs, chors, chorm, ppndh, igps and ans.

### Metabolic control analysis

The data acquired during metabolic analysis with shikimate as additional perturbation substrate reveals detailed insights into the cells’ metabolism during L-tryptophan production from glycerol and provides important information for the modelling of intracellular flux distributions and for thermodynamic analysis of the metabolic network. Nonetheless, due to the complexity of the considered pathways, identifying targets for the genetic strain optimisation is challenging. Local and global coefficients, which mathematically represent metabolic connections, offer the possibility of quantifying the changes in steady-state fluxes and metabolite concentrations in response to perturbations and this enables the quantitative description of metabolic control [59]. In this work, MCA is applied to estimate local elasticities, which describe the sensitivity of a single enzyme towards a change in individual metabolite levels and global control coefficients (FCC), which are a measure of how a steady-state flux is affected by a change in enzyme levels. Depending upon the thermodynamic state of a reaction, the elasticities are derived either directly from Gibbs reaction energies and stoichiometry, when operating close to equilibrium or by definition of effectors and applying the lin-log approach (see chapter 2.9), when Δ_r_G’ < −10 kJ mol^−1^. Control coefficients were estimated via a linearisation approach by Visser and Heijnen [59], and they are defined relative to a reference steady state, in this case, the L-tryptophan fed-batch production process during metabolic analysis. Through normalisation towards the reference steady-state, coefficients become dimensionless. Data analysis and the results of thermodynamic flux analysis and elasticities enabled the estimation of FCCs for the considered pathways. FCCs describe the effect of one per cent change in a single enzyme level on a specific flux in the metabolic network.

**Fig. 7:**
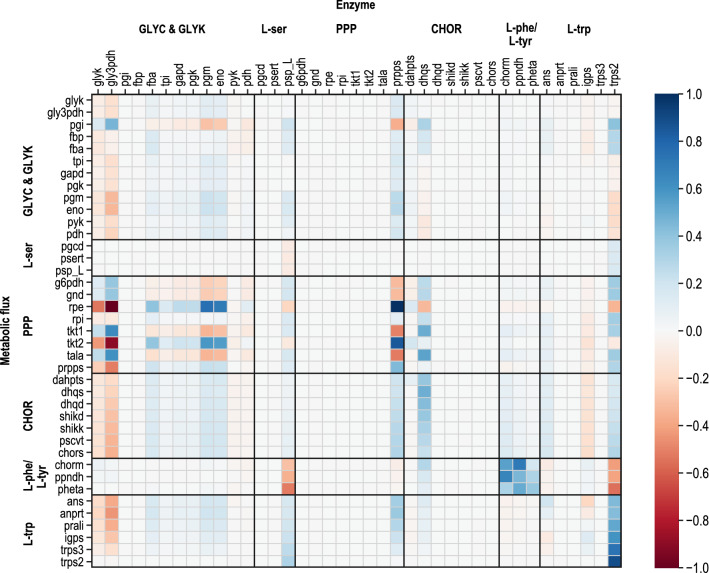
Mean flux control coefficients (unitless) estimated by metabolic control analysis of L-tryptophan production with *E. coli*. Rows represent enzyme activities, and lines refer to metabolic fluxes. The X-axis represents enzyme capacity; corresponding metabolic fluxes are described on the y-axis. The effects of changes in enzyme activity by one per cent are illustrated

Consequently, values between −1.0 and 1.0 were obtained. Activating effects are represented by positive coefficients, inhibiting effects by negative values. In Fig. 7, relevant FCCs are depicted for the considered metabolic network. In the following, significant results of MCA are described with a particular focus on FCCs of direct relevance for L-tryptophan production.

Both enzymes of glycerol degradation (glyk and gly3pdh) have a negative control on fluxes of glycolysis, gluconeogenesis and glycerol metabolism, except for pgi. A positive control regarding fluxes of the PPP was estimated. This control appears due to the competing redistribution of carbon fluxes between PPP and upper glycolysis. Both chorismate and L-tryptophan biosynthesis are negatively controlled by glyk and gly3pdh, indicating that a higher substrate supply does not lead to enhanced fluxes in these pathways. This can be attributed to the measured decrease in L-tryptophan production rates, despite increasing substrate uptake rates. Possibly high DHAP concentrations trigger the activation of methylglyoxal-pathway when glycerol is used as substrate [66]. This might cause a restriction of L-tryptophan biosynthesis. Contradicting results were published by Cintolesi et al. [67] for anaerobic glycerol degradation, where positive FCCs on glycolysis were found. The glycolytic enzymes fba, pgm and eno have minor positive control over fluxes in chorismate and L-tryptophan biosynthesis, which is related to positive effects on chorismate and L-tryptophan production by the improvement of PEP supply. This has also been reported by Weiner [55]. The slight negative control of pyk in relation to chorismate biosynthesis might be correlated to the competitive usage of PEP. This finding coincided with previously published results by Tröndle et al. [17] for L-tryptophan producing cells and was also described by Weiner et al. [2] during L-phenylalanine production with *E. coli*.

In the L-serine biosynthesis pathway, only phosphoserine phosphatase (psp_L) has controlling effects. Positive FCCs were received for the dephosphorylating enzyme towards all the reactions that are directly or indirectly connected with L-tryptophan biosynthesis, and highly negative FCCs were estimated for fluxes from chorismate to L-phenylalanine. The highest control was estimated for tryptophan synthase (trps2). Accordingly, an L-serine supply enhancement would lead to increased L-tryptophan production but decreased by-product synthesis. This contrary effect is linked to the L-serine consumption in L-tryptophan biosynthesis, where indole and L-serine are converted to L-tryptophan. L-serine is not used for L-phenylalanine biosynthesis, and through improving L-serine provision, fluxes starting from chorismate are shifted in the direction of L-tryptophan production while by-product formation is decreased. This result agrees with Tröndle [17].

The common PPP, including the reactions glucose 6-phosphate dehydrogenase (g6pdh), phosphogluconate dehydrogenase (gnd), rpe, rpi, tkt1, tkt2 and tala does not exercise control on any flux in the considered metabolic network, which can be explained by their thermodynamic state that is characteristically close to equilibrium. In a continuation of the classic PPP, prpps catalyses the reaction, where PRPP is generated from R5P. A strong positive control of prpps for all fluxes involved in L-tryptophan biosynthesis was found, since PRPP acts as a direct precursor metabolite. In correspondence to this, L-phenylalanine biosynthesis from chorismate is negatively influenced by prpps. Here, the results also coincide with Tröndle [17].

Among the enzymes participating in chorismate biosynthesis, only dhqs has control over reactions in the metabolic network, according to the MCA results. The Gibbs reaction energy of dhqs was estimated to be highly negative, indicating an active regulation of its enzymatic capacity and suggesting a controlling potential of the reaction. Even though dhqs was previously identified as a limitation in shikimate biosynthesis [68, 69], it has not been determined as a controlling step in previous MCAs of L-tryptophan production with *E. coli*. Control of dhqs on L-tryptophan biosynthesis reaches down to trps3, while L-serine deficiency attenuates the effect on trps2.

Enzymes of the biosynthesis of the aromatic by-products have high positive control over themselves and slightly negative control towards fluxes producing L-tryptophan, due to the competitive consumption of chorismate of both branches. Since only very low L-phenylalanine and L-tyrosine formation rates and concentrations were measured, the controlling effect of these enzymes on L-tryptophan formation is relatively small. Presumably, a further downregulation of by-product formation will have little impact on productivity towards L-tryptophan.

Enzymes participating in L-tryptophan production starting from chorismate have controlling effects on the central metabolism, but these enzymes mainly impact L-tryptophan formation itself. For anthranilate synthase (ans) a positive control was received for the PPP and chorismate synthesis on itself and the following flux of anprt, whereas a slightly negative control was estimated for fluxes of igps and trps3. The resulting positive control of ans on igps complies well with earlier findings of non-competitive feed-forward inhibition of igps by ANTH [24]. The far-reaching effect on PPP and chorismate synthesis arises from the connection through PRPP, which is supplied by the PPP and is used as precursor metabolite by the reaction catalysed by ans. The opposite effects were obtained for igps, which can be explained by the connection through ANTH, a defined effector for the enzyme igps in MCA [70]. The strongest positive control is exercised by trps2 on reactions of the L-tryptophan biosynthesis. In particular, the control towards itself is highly pronounced. Thus, highly negative control of this reaction towards the by-product formation was obtained. According to the estimations, trps2 positively controls the PPP and chorismate biosynthesis. For glycolysis, the estimated control was partly positive and negative.

As is apparent from the results of MCA discussed above, metabolic control does not occur at one single point in the metabolism, but there is a distribution of FCCs over the whole considered network. Several enzymes are contributing to the overall control, and limitations arise at multiple sites in the metabolism. Kacser and Burns [7] firstly described this effect of metabolic control. This work focuses on metabolic control on the L-tryptophan biosynthesis pathway, where several controlling sites were determined. In glycerol metabolism, highly negative FCCs were found for the enzymes glyk and gly3pdh. A positive control of psp_L, the last enzyme of L-serine biosynthesis and prpps indicate a deficiency in precursor supply, which leads to limitations in L-tryptophan productivity. Targeted deflection of cell metabolism within chorismate biosynthesis was achieved via feeding of shikimate as an additional perturbation substrate. The enhanced perturbation in this particular metabolic pathway enabled the identification of dhqs as a limiting step for L-tryptophan biosynthesis. Metabolic control in L-tryptophan biosynthesis was most pronounced for trps2, catalysing the synthesis of L-tryptophan from indol and L-serine. Furthermore, a slight control was determined for igps.

### Relative expression analysis of targeted genes

**Fig. 8:**
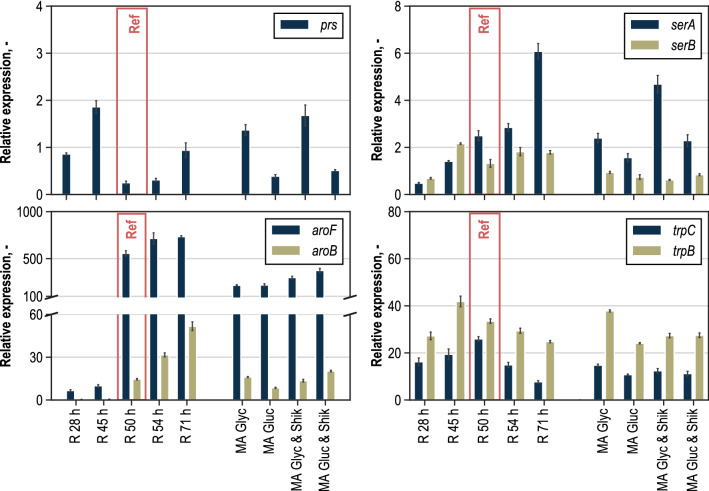
Relative gene expression (without unit) of genes *prs*, *serA*, *serB*, *aroF*, *aroB*, *trpC* and *trpB*, relative to *ftsZ* gene of samples from reference L-tryptophan production process after 28 h (R 28 h), 45 h (R 45 h), 50 h (R 50 h), 54 h (R 54 h) and 71 h (R 71 h) process time (IPTG for the induction of inducible genes was added 45 h after inoculation) and from analysis reactors with glycerol (MA Glyc), glucose (MA Gluc), glycerol and shikimate (MA Glyc & Shik) as well as glucose and shikimate (MA Gluc & Shik)

The results of MCA provided information on possible starting points for genetic strain optimisation. To further investigate the potential limitations in enzymatic capacity, a relative expression of selected genes of interest was analysed by RT-qPCR. Figure 8 shows results for the genes *prs*, *serA*, *serB*, *aroF*, *aroB*, *trpC* and *trpB.* MCA revealed a strong positive control of prpps on the entire metabolic network. The corresponding gene *prs* was expressed significantly higher before induction. Relative expression was at its minimum at the time point immediately after IPTG addition. Comparably low expressions were measured in both analysis reactors when glycerol was used for perturbation. In the analysis reactors where glucose was supplied as perturbation substrate, the gene expressions were more than twice as high as in the reactors with glycerol. In those cases where shikimate was added as secondary perturbation substrate, expression of the *prs* gene was slightly higher than without addition. Towards the end of the process, the relative expression increased so that it reached again the level measured at a process time of 28 h. Low expression of the *prs* gene during metabolic analysis correlate with the results from MCA. Possibly, a downregulation of *prs* during the production phase may lead to constraints in enzymatic capacity of prpps, which in turn limit PRPP supply for L-tryptophan biosynthesis.

High metabolic control on L-tryptophan production was also determined in L-serine supply. The enzyme psp_L, encoded by the *serB* gene, was estimated to be a limiting step for L-tryptophan formation in the considered metabolic model. In contrast to *serB* gene, which is not overexpressed in the *E. coli* strain used, *serA* gene is already integrated chromosomally in extra copies. More precisely, the gene inserted, encodes for a feed-back-resistant phosphoglycerate-3-phosphate dehydrogenase (*serA*^FBR^) to circumvent inhibition by L-serine. A comparison of the relative expressions of *serA* and *serB* shows that whereas the relative expression of *serA* steadily increases, the *serB* expression dropped after induction and stagnated afterwards. The relative expression of *serA* during metabolic analysis was similarly high compared to the reference state. However, the relative expression was significantly increased in the analysis reactor with glycerol and shikimate as perturbation substrates. Relative expression of *serB* during metabolic analysis was slightly lower compared to the reference state. Unlike the expression of *serA*, the expression of *serB* dropped after induction and did not increase significantly until the end of the process. Possibly, the unbalanced expression of genes in L-serine biosynthesis pathway may lead to disparities between reaction fluxes and consequently, limitations occur. An increase in *serB* expression might compensate for these imbalances.

In chorismate biosynthesis pathway, *aroB* was identified as a limiting step for L-tryptophan production by MCA, even though the gene is overexpressed together with *aroF* and *aroL* on the plasmid in the *E. coli* strain used. Figure 8 shows the relative expression of *aroF* and *aroB* comparison. An abrupt rise in the relative expression of *aroF* from 6.6 before induction to 556.8 immediately after IPTG addition was measured. During the continuing process, a constant relative expression of *aroF* was observed. In samples taken from the analysis reactors during metabolic analysis, the relative expression of *aroF* was lower than the reference state. Relative expression of *aroB* below 1 was observed until a process time of 45 h. After induction, the expression steadily increased until the end of the process, where a relative expression of 51.7 was reached. Similar relative expressions were measured in the sample from the reference process state and the analysis reactors. At the timepoint of metabolic analysis, the expression of *aroF* is 20-fold higher than the expression of *aroB* in the sample from the reference state. In the analysis reactors, the relative expression disparity varies from 13 to 26-fold between *aroF* and *aroB.* Although the relative expression of *aroF* reaches its maximum level immediately after induction, the upregulation of *aroB* lags behind and is weakened compared to *aroF.* The delayed rise in the relative expression of *aroB* might be the reason for emerging limitations within chorismate biosynthesis. The differences in the expressions are probably a consequence of the arrangement of the genes in the expression cassette on the plasmid, where *aroF* directly follows up the promotor.

Further limitations were estimated in L-tryptophan biosynthesis itself. The strongest control was found for trps2, and igps was also identified to slightly control L-tryptophan formation. The corresponding genes for these limiting enzymes are *trpB* and *trpC*, respectively. The relative expressions are depicted in Fig. 8. The expression of both genes increases until the time point of induction and drops again after IPTG was added. The expression of *trpB* is higher than that of *trpC* in both the cells from the production process and the cells from the analysis reactors. Due to the competing expression of many inducible genes after IPTG addition, the metabolic machinery of *E. coli* operates at full capacity, and this might be the reason for the downregulation of both genes after induction.

## Conclusions

The application of thermodynamics-based flux analysis [14] enabled estimations of flux distributions as a function of thermodynamic constraints and intracellular metabolite concentrations. Through the association of thermodynamics with flux variability analysis, misconceptions regarding the direction of metabolic reactions could be ruled out. Furthermore, the newly applied sampling function [58] enabled the statistical evaluation of the solution spaces. These developments improved the accuracy and predictiveness of computations without altering the fundamental results of MCA.

Enhanced perturbation in chorismate biosynthesis induced by an external shikimate supply, clearly showed that dhqs holds positive control over reactions of chorismate- and L-tryptophan biosynthesis, reaching down to trps3. A previously unknown controlling enzyme was identified from the intensified deflections emerging from shikimic acid. Transcriptome analysis of the corresponding genes showed disparities in gene expression of the *aroB* and *aroF* genes, which are most likely responsible for the limitations occurring in chorismate biosynthesis. Therefore, the insertion of additional copies of *aroB* gene, in combination with the resolution of other limitations (e.g., improved supply of PRPP, and psp_L, respectively) might lead to improved productivity.

Surprisingly, the expressions of the *prs*, *trpC* and *trpB* genes encoding the enzymes prpps, igps and trps2, which were estimated by MCA to limit L-tryptophan production, were downregulated after induction with IPTG. It seems that the expression of the inducible genes is boosted by induction with IPTG at the expense of the transcription of other genes. Consequently, overexpression of the *prs*, *trpB* and *trpC* limiting genes or the use of alternative strong promoters, will probably help to tackle the bottlenecks and reroute the carbon flow from by-product formation to L-tryptophan biosynthesis. Due to the close connection to energy metabolism via ATP dephosphorylation and PRPP’s contribution in salvage metabolism [71], genetic modifications relating to prpps might be difficult.

In L-serine biosynthesis, psp_L was estimated to control L-tryptophan formation during metabolic analysis. Results of the expression analysis revealed differing expression levels of the *serA* and *serB* genes, which might be the reason for unbalanced fluxes and consequential internal constraints in L-serine biosynthesis. Limitations could be avoided by overexpressing psp_L or by reducing competing reactions. The negative control of glycerol degradation (enzymes glyk and gly3pdh) on chorismate and L-tryptophan production might be attributed to the toxic intracellular accumulation of DHAP. Further research is necessary to analyse these limitations in detail and devise possible solutions.

## Supplementary Information

Below is the link to the electronic supplementary material.

Supplementary file1 (PDF 64 KB)

## Data Availability

Not applicable.
