# Supplementary material for: Metabolic control analysis of L-tryptophan producing Escherichia coli applying targeted perturbation with shikimate
Source: Bioprocess Biosyst Eng. 2021 Sep 14;44(12):2591–613. doi: 10.1007/s00449-021-02630-7 (PMC8536597; doi:10.1007/s00449-021-02630-7)
Supplement: Supplementary file 1 — Supplementary file1 (PDF 64 KB) [file 449_2021_2630_MOESM1_ESM.pdf]

Bioprocess and Biosystems Engineering

Metabolic Control Analysis of L-tryptophan producing *Escherichia coli* applying targeted perturbation with shikimate

Kristin Schoppel[1], Natalia Trachtmann[2], Fabian Mittermeier[1], Georg A. Sprenger[2], Dirk Weuster-Botz[1]

[1] Technical University of Munich, Institute of Biochemical Engineering, Boltzmannstraße 15, 85748 Garching, Germany

[2] University of Stuttgart, Institute of Microbiology, Allmandring 31, 70569, Stuttgart, Germany

Email: dirk.weuster-botz@tum.de

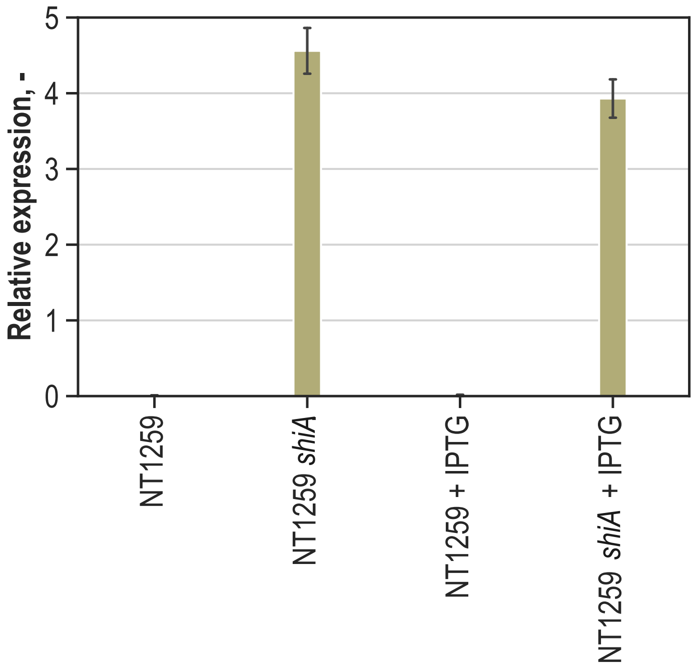

Supplementary Fig. 1: Relative gene expression (without unit) of *shiA* gene relative to *ftsZ* gene of *E. coli* NT1259 pF112*aroFBL*$_{\mathrm{Kan}}$ (NT1259) and *E. coli* NT1259 *shiACg* pF112*aroFBL*$_{\mathrm{Kan}}$ (NT1259 *shiA*) after 17 h cultivation in 50 mL lysogeny broth in shake-flasks without and with 0.5 mM IPTG at 30°C and 200 rpm shaking.

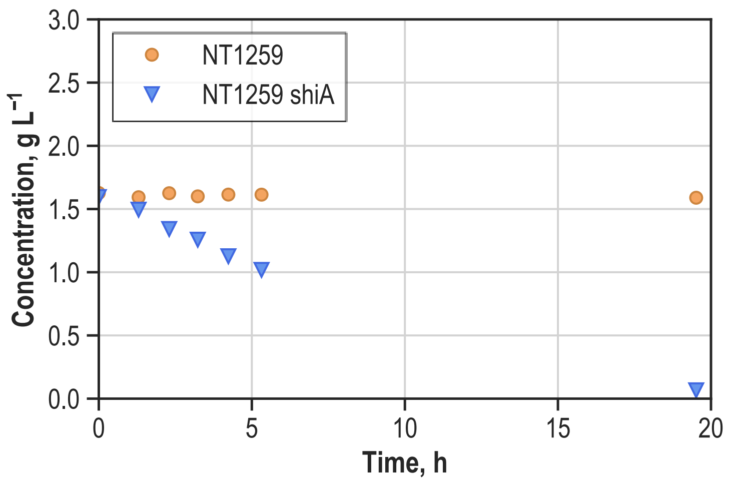

Supplementary Fig. 2: Shikimate concentrations during cultivation of *E. coli* NT1259 pF112*aroFBL*$_{Kan}$ (NT1259) and *E. coli* NT1259 *shiACg* pF112*aroFBL*$_{Kan}$ (NT1259 *shiA*) in 100 mL minimal medium (6 g L^{-1} glycerol and 1.6 g L^{-1} shikimate, 0.3 mM IPTG) in shake-flasks at 37°C and 200 min^{-1} shaking.
